# New Chemo-, Regio- and Stereoselective Reactions and Methods in Organic Synthesis

**DOI:** 10.3390/ijms252413409

**Published:** 2024-12-14

**Authors:** Maxim V. Musalov

**Affiliations:** A. E. Favorsky Irkutsk Institute of Chemistry, Siberian Division of the Russian Academy of Sciences, 1 Favorsky Str., Irkutsk 664033, Russia; musalov_maxim@irioch.irk.ru

The Special Issue “New Chemo-, Regio- and Stereoselective Reactions and Methods in Organic Synthesis” collects eight articles that have developed advanced approaches to the chemo-, regio- and stereoselective synthesis of novel important compounds, scaffolds, synthons, and practically valuable products.

The introduction of new reagents, selenium dihalides, into organoselenium synthesis has opened up effective approaches to new classes of organoselenium compounds [1,2,3,4]. This area of organic chemistry based on these bifunctional electrophilic selenium reagents continues to develop actively. For example, selenium dihalides have made it possible to synthesize a number of 9-selenabicyclo[3.3.1]nonane derivatives with high regio- and stereoselectivity, which have proven themselves not only as convenient and accessible model compounds but also as non-toxic modulators of the antioxidant profile, increasing both glutathione peroxidase and glutathione reductase activities in vivo [5,6]. An efficient stereoselective synthesis of the 9-selenabicyclo[3.3.1]nonene-2 derivatives, a new class of organoselenium heterocyclic compounds and prospective intermediates for organic synthesis, based on the remarkable reaction of 2,6-dibromo-9-selenabicyclo[3.3.1]nonane with acetonitrile, was developed by Potapov et al. (contribution 1). Azido-, amino-, and alkoxy-9-selenabicyclo[3.3.1]nonene-2 derivatives were obtained in 88–99% yields based on the azidation and alkoxylation reactions of 6-bromo-9-selenabicyclo[3.3.1]nonene-2. The methods of amination of 2,6-dibromo-9-selenabicyclo[3.3.1]nonane are characterized by simple reaction conditions, broad-scope reactions, and convenient isolation procedures. The products as dihydrobromide salts were isolated from the reaction mixture by simple filtration. The diamino derivatives as free bases were also obtained in high yields directly from 2,6-dibromo-9-selenabicyclo[3.3.1]nonane, and these compounds did not require additional purification. This method meets the criteria of click chemistry. The high GPx-mimetic activity for water-soluble dihydrobromide derivatives, containing bis-diethylamino, bis-isopropylamino, bis-phenylamino, and bis-pyrrolidinyl substituents, was shown.

Diseases such as Alzheimer’s disease, epilepsy, depression, etc., pose significant challenges for the synthesis of new potential drugs. One of the causes of these diseases is the disruption of AMPA receptor activity. It is known that positive allosteric modulators (PAM) of the AMPA receptor, binding to its allosteric binding site, allow fine-tuning the AMPA receptor signaling, as well as avoiding excitotoxicity problems usually observed when administering a direct agonist in excessive doses [7,8,9]. On the other hand, its negative allosteric modulators (NAM) may have antiepileptic and neuroprotective properties. Isoxazoles are a promising structural core for the synthesis of bivalent AMPA ligands [10,11].

An efficient synthesis of a series of new functionalized isoxazoles containing aromatic or aliphatic linkers was developed by Palyulin et al. (contribution 2). In this work, an approach based on the linking of two functional isoxazole cores by binucleophilic O,O-N,N-S,S-linkers was implemented. Electrophysiological experiments demonstrated that the obtained new compounds exhibited activity as positive allosteric modulators of the AMPA receptor. A number of compounds were found to be effective negative modulators of the receptor, forming interesting activity cliffs.

The aromaticity and reactivity of heterocyclic compounds are among the most studied problems in organic chemistry. The properties of fused heterocyclic systems often face a number of problems related to their aromaticity/antiaromaticity and the resulting differences in reactivity [12,13]. Heterocycles with high electron affinity as electron-withdrawing blocks have received much attention [14,15]. A special place among these heterocycles is occupied by 2,1,3-benzothiadiazole (BTD) and its derivatives, e.g., benzo[1,2-c:4,5-c0]bis[1,2,5]thiadiazole (BBT), due to their excellent properties such as strong electron-withdrawing properties, intense light absorption, and good photochemical stability [16]. Furthermore, replacing the 1,2,5-thiadiazole ring in 2,1,3-benzothiadiazole (BTD) with a 1,2,3-thiadiazole core results in benzo[d][1,2,3]thiadiazole (isoBTD) compounds with properties similar to those of BTD but with higher ELUMO and band gap (Eg) values [17]. The BBT isomer, benzo[1,2-d:4,5-d0]bis([1,2,3]thiadiazole) (isoBTD), also exhibits promising electron acceptor properties, and its 4,8-dibromo derivative can be successfully involved in aromatic nucleophilic substitution reactions and in palladium-catalyzed Suzuki–Miyaura and Stille cross-coupling reactions with selective formation of mono- and bis-arylated heterocycles [18]. It is known that many sulfur-containing heterocycles have anticancer activity [19], due to the low-lying C-S σ* or C-N σ* orbitals, which are responsible for drug–target interactions [20]. Therefore, isoBBT derivatives are of additional interest in terms of potential biological activity.

Rakitin et al. confirmed the aromaticity of benzo[1,2-d:4,5-d0]bis([1,2,3]thiadiazole) (isoBBT) and benzo[1,2-c:4,5-c0]bis [1,2,5]thiadiazole (BBT) heterocycles by two modern criteria, EDDB and GIMIC, at the MP2 level of theory and additionally by UV–Vis spectroscopy (contribution 3). A significant difference in the aromaticity degree of isoBBT and BBT was shown, which significantly affected the reactivity of these compounds. Based on the study of nucleophilic aromatic substitution reactions in bromo derivatives of isoBBT, a number of 4-amino- and 4-thio substituted products, 4-arylisoBBT derivatives, inaccessible by other methods, were obtained by several versions of palladium-catalyzed cross-coupling, namely, the Suzuki and Stille reactions and direct C–H arylation with 2-unsubstituted thiophenes. Selective methods for the synthesis of 4-bromo-8-arylisoBBT derivatives via the cross-coupling reactions of 4-bromoisoBBT with (het)aryl halides and oxidative direct C–H arylation with thiophenes were also developed.

It is known that the biological activity of compounds such as isoindolinones and pyrazoles [21,22] varies significantly depending on the linking of additional nitrogen-containing substituents or heterocyclic groups such as piperazines and piperidines. Therefore, the development of new synthetic methods to furnish new hybrid molecules is of paramount importance, even though additional difficulties can be encountered, especially when new stereocenters are formed. In fact, asymmetric construction of the isoindolinone ring is a challenging research field, and many groups are involved [23]. Massa et al. have developed a number of approaches to the asymmetric synthesis of 3-substituted [24] and 3,3-disubstituted isoindolinones [25], developing new cascade reactions, often using organocatalytic systems and producing several new enantioenriched products. Some of the obtained isoindolinones were also used in the general synthesis of biologically significant compounds [24]. As the development of these studies, a method for the asymmetric synthesis of new heterocyclic hybrid molecules with the use of acetylacetone as a nucleophile in an asymmetric reaction with N-carbamoyl-α-amidosulfone in the presence of Takemoto’s bifunctional organocatalyst was discovered (contribution 4). The article discusses studies on the reactivity of the obtained semi-products, the effect of the environment and temperature on the rate and selectivity of the process, and the effectiveness of various catalysts on the enantioselectivity of the process. As a result, isoindolinone substituted in the 3-position by a pyrazole ring was obtained from acetylacetone with high enantioselectivity (89% ee) and an excellent yield of about 70% for four stages of synthesis. It was shown that the Takemoto organocatalyst was more effective in comparison with other catalytic systems in these reactions. It was also established that the bifunctional nature of the catalysts also promotes lactamization, which was confirmed through a series of control experiments.

Pyrroloimidazoles are important structural motifs in a number of natural products and bioactive molecules and key components for optoelectronic devices [26,27]. The known approaches to pyrrolo[1,2-c]imidazoles include the intermolecular cyclocondensation of pyrrole, isocyanate, and phosgene or thiophosgene [28]; the reaction of benzotriazol-1-yl(1H-pyrrol-2-yl)methanone with ketone, isocyanate, or isothiocyanate [29]; Rh(III)- and Pd(0)-catalyzed C-H functionalization of pyrroles with alkynes, alkenes, and diazo compounds [30]; the post-Ugi cascade reaction [27]; and the flash vacuum pyrolysis of imidazol-1-ylacrylates [31].

One of the newest approaches to the assembly of dipyrrolo[1,2-a:1′,2′-c]imidazole core was discovered by Trofimov et al., based on accessible acylethinylpyrroles (contribution 5) [32]. The efficient and convenient method for the synthesis of previously unknown pyrrolo[1′,2′:2,3]imidazo[1,5-a]indoles and cyclohepta[4,5]pyrrolo[1,2-c]pyrrolo[1,2-a]imidazoles was developed via the reaction of non-catalytic [3 + 2]-annulation. In this work, the effect of the reaction conditions on the chemo- and regioselectivity of the process was studied, and the results of the screening of reaction conditions were discussed using the example of the benzoylethynyltetrahydroindole-pyrroline model system. Optimization of the proposed synthetic method allowed implementing a catalyst-free one-pot chemo- and regioselective synthesis of polycondensed indoles and imidazoles functionalized with acylethenyl groups in up to 81% yields under mild conditions. It was found that the physicochemical properties of the new compounds, which are of crucial importance for TADF emitters in OLEDs, taking into account their availability, allow the use of such compounds in optoelectronics. The developed approach also opens up a straightforward route to biochemically related functionalized polycondensed heterocyclic systems, a prospective platform for drug discovery.

Selective serotonin reuptake inhibitors (SSRIs) are widely used in the treatment of depression and other mental disorders, although current treatment methods suffer from a number of pharmacological drawbacks [33]. The development of synthetic methods for the preparation of analogues of 5-hydroxytryptamine and homotryptamine are the subject of research targeting new drugs that act on the central nervous system through the serotonergic mechanism and exhibit multidirectional pharmacological activity [33,34]. Of particular interest are derivatives with a conformationally restricted aminoethyl residue at position 3 of the indole ring. Conformational restrictions of the side chain are known to have a significant effect on the biological activity of compounds that inhibit the serotonin transporter protein SERT and the 5-HT1A receptor [35,36]. Recently, interest in new ligands with high binding to the 5-HT_6_ receptor has increased significantly due to the role of this receptor in the pathomechanisms of depression, schizophrenia, and Alzheimer’s and Parkinson’s diseases [37].

A method for the preparation of a series of chiral derivatives of (R)- and (S)-3-(piperidin-3-yl)-1H-indole based on racemic mixtures of 3-(piperidin-3-yl)-1H-indole and its derivatives was proposed by Slifirski et al. (contribution 6). The synthetic method significantly simplified the separation of diastereomers and allowed the isolation of pure enantiomers of the final products, which are of crucial importance for the development of new drugs. In the course of the studies, the authors unexpectedly obtained new tetracyclic derivatives of 9,12-diazatetracyclo[10.3.1.0^2,10^.0^3,8^]hexadeca-2(10),3(8),4,6-tetraenes. In addition, the authors proved the absolute structures of all the obtained compounds.

The synthesis of large and medium-sized carbo- and heterocycles is largely based on ring expansion reactions. A number of approaches have been developed for the synthesis of a wide variety of classes of compounds, including lactones, O-, N-, S-heterocyclic rings, substituted azulenes, and various azepine derivatives. Ring expansion reactions can be based on various substrates and proceed by different mechanisms, which include a number of rearrangements and cycloaddition reactions [38,39,40]. A wide range of reactions that demonstrate the synthetic importance of functionalized carbocyclic and heterocyclic molecules formed via six-membered ring expansion reactions, based on the literature published from 2017 to 2022, are presented and thoroughly discussed in the review by Zaki et al. (contribution 7). The review was structured according to the final reaction products and discussed methods for the synthesis of lactams from cycloketones, nitroso compounds, β-keto esters, and oximes; the synthesis of lactones based on oxazines, the reactions of lactones with azeridines, and the synthesis of azulenes based on chromenones, derivatives of 1,3-diazepinones from functional 1,4-dihydro-2H-pyrimidinones; and the synthesis of tropone derivatives from alkynyl quinols. It is worth noting that the authors provide the results of the biological activity studies of the products. The review also discusses such reactions as Aza–Claisen rearrangement [38], Beckmann rearrangement [39], Tiffeneau–Demjanov rearrangement [40], Schmidt rearrangement [41], [10+4] and [6+6] cycloaddition reactions [41,42].

At present, chemical syntheses using natural metabolism attract much attention from scientists. A wide range of natural carbohydrates serves as a starting point for the chemical transformation of biomass, which leads to bioderivatives of furans. Currently, the synthesis and application of furan compounds is an important branch of green and sustainable chemistry. Indeed, many articles describe the synthesis of 5-HMF and its derivatives, as well as the preparation of new materials based on them [43,44,45]. At the same time, 1,4-functionalized C6-furan fragments can be found among the natural compounds. The search for practically significant natural products with subsequent biotechnological production is an attractive methodology for a sustainable development [46]. The number of oxygen atoms in the C6-furanic derivatives can be the key factor for their practical possibilities [47,48]. A discussion of the literature data on natural compounds that can be the target of full-cycle methods or biological studies was given in the excellent review by Ananikov et al. (contribution 8). The authors proposed the clear and well-structured review of 1,4-disubstituted furan derivatives based on the oxidation level of the initial 1,4-dimethylfuran to 5-formylfuran-2-carboxylic acid and furan-2,5-dicarboxylic acid. The review discussed methods for the synthesis and sequential functionalization of 1,4-dimethylfuran based on increasing the oxidation level of the initial compounds, which clearly demonstrates the possibilities of implementing biosimilar synthetic approaches to the synthesis of practically significant furanyl compounds. The review of natural biosynthetic pathways and their incorporation into practice is of great importance for sustainable chemistry.

## References

[1] Potapov V.A., Amosova S.V., Belozerova O.V., Albanov A.I., Yarosh O.G., Voronkov M.G. (2003). Synthesis of 3,6-dihalo-4,4-dimethyl-1,4-selenasilafulvenes. Chem. Heterocycl. Compd..

[2] Martins G.M., Back D.F., Kaufman T.S., Silveira C.C. (2018). SeCl2-Mediated Approach Toward Indole-Containing Polysubstituted Selenophenes. J. Org. Chem..

[3] Arsenyan P. (2014). A simple method for the preparation of selenopheno[3,2-*b*] and [2,3-*b*]thiophenes. Tetrahedron Lett..

[4] Abakumov G.A., Piskunov A.V., Cherkasov V.K., Fedushkin I.L., Ananikov V.P., Eremin D.B., Gordeev E.G., Beletskaya I.P., Averin A.D., Bochkarev M.N. (2018). Organoelement chemistry: Promising growth areas and challenges. Russ. Chem. Rev..

[5] Accurso A.A., Cho S.-H., Amin A., Potapov V.A., Amosova S.V., Finn M.G. (2011). Thia-, Aza-, and Selena[3.3.1]bicyclononane Dichlorides: Rates vs Internal Nucleophile in Anchimeric Assistance. J. Org. Chem..

[6] Musalov M.V., Kapustina I.S., Spiridonova E.V., Ozolina N.V., Amosova S.V., Borodina T.N., Potapov V.A. (2023). Transannular Selenocyclofunctionalization of 1,5-cyclooctadiene: The Antioxidant Properties of 9-selenabicyclo[3.3.1]nonane Derivatives and the Discovery of Increasing Both GPx and GR Activities. Inorganics.

[7] Brogi S., Campiani G., Brindisi M., Butini S. (2019). Allosteric modulation of ionotropic glutamate receptors: An outlook on new therapeutic approaches to treat central nervous system disorders. ACS Med. Chem. Lett..

[8] Lee K., Goodman L., Fourie C., Schenk S., Leitch B., Montgomery J.M., Donev R. (2016). AMPA receptors as therapeutic targets for neurological disorders. Ion Channels as Therapeutic Targets, Part A.

[9] Reuillon T., Ward S.E., Beswick P. (2016). AMPA receptor positive allosteric modulators: Potential for the treatment of neuropsychiatric and neurological disorders. Curr. Top. Med. Chem..

[10] Golubeva E.A., Lavrov M.I., Radchenko E.V., Palyulin V.A. (2023). Diversity of AMPA receptor ligands: Chemotypes, binding modes, mechanisms of action, and therapeutic effects. Biomolecules.

[11] Vasilenko D.A., Sadovnikov K.S., Sedenkova K.N., Karlov D.S., Radchenko E.V., Grishin Y.K., Rybakov V.B., Kuznetsova T.S., Zamoyski V.L., Grigoriev V.V. (2021). A facile approach to bis(isoxazoles), promising ligands of the AMPA receptor. Molecules.

[12] Krygowski T.M., Cyra´nski M.K. (2009). Aromaticity in Heterocyclic Compounds. Topics in Heterocyclic Chemistry.

[13] Martín N., Scott L.T. (2015). Challenges in aromaticity: 150 years after Kekulé’s benzene. Chem. Soc. Rev..

[14] Wu Y., Zhu W.-H., Zakeeruddin S.M., Grätzel M. (2015). Insight into D–A–π–A Structured Sensitizers: A Promising Route to Highly Efficient and Stable Dye-Sensitized Solar Cells. ACS Appl. Mater. Interfaces.

[15] Zhang Y., Song J., Qu J., Qian P.-C., Wong W.-Y. (2021). Recent progress of electronic materials based on 2,1,3-benzothiadiazole and its derivatives: Synthesis and their application in organic light-emitting diodes. Sci. China Chem..

[16] Rakitin O.A. (2019). Recent Developments in the Synthesis of 1,2,5-Thiadiazoles and 2,1,3-Benzothiadiazoles. Synthesis.

[17] Gudim N.S., Knyazeva E.A., Mikhalchenko L.V., Golovanov I.S., Popov V.V., Obruchnikova N.V., Rakitin O.A. (2021). Benzothiadiazole vs. iso-Benzothiadiazole: Synthesis, Electrochemical and Optical Properties of D–A–D Conjugated Molecules Based on Them. Molecules.

[18] Chmovzh T.N., Alekhina D.A., Kudryashev T.A., Rakitin O.A. (2022). Efficient Synthesis of 4,8-Dibromo Derivative of Strong Electron-Deficient Benzo[1,2-d:4,5-d’]bis([1,2,3]thiadiazole) and Its SNAr and Cross-Coupling Reactions. Molecules.

[19] Cascioferro S., Petri G.L., Parrino B., Carbone D., Funel N., Bergonzini C., Mantini G., Dekker H., Geerke D., Peters G.J. (2020). Imidazo[2,1-b] [1,3,4]thiadiazoles with antiproliferative activity against primary and gemcitabine-resistant pancreatic cancer cells. Eur. J. Med. Chem..

[20] Beno B.R., Yeung K.-S., Bartberger M.D., Pennington L.D., Meanwell N.A. (2015). A Survey of the Role of Noncovalent Sulfur Interactions in Drug Design. J. Med. Chem..

[21] Upadhyay S.P., Thapa P., Sharma R., Sharma M. (2020). 1-Isoindolinone scaffold-based natural products with a promising diverse bioactivity. Fitoterapia.

[22] Kamal R., Kumar A., Kumar R. (2023). Synthetic strategies for 1,4,5/4,5-substituted azoles: A perspective review. J. Heterocycl. Chem..

[23] Di Mola A., Palombi L., Massa A., Attanasi O.A., Noto R., Spinelli D. (2014). An Overview on asymmetric synthesis of 3-substituted isoindolinones. Targets in Heterocyclic Systems.

[24] Di Mola A., Tiffner M., Scorzelli F., Palombi L., Filosa R., de Caprariis P., Waser M., Massa A. (2015). Bifunctional Phase-Transfer Catalysis in the Asymmetric Synthesis of Biologically Active Isoindolinones. Beilstein J. Org. Chem..

[25] Scorzelli F., Di Mola A., De Piano F., Tedesco C., Palombi L., Filosa R., Waser M., Massa A. (2017). A systematic study on the use of different organocatalytic activation modes for asymmetric conjugated addition reactions of isoindolinones. Tetrahedron.

[26] Yamawaki I., Matsushita Y., Asaka N., Ohmori K., Nomura N., Ogawa K. (1993). Synthesis and aldose reductase inhibitory activity of acetic acid derivatives of pyrrolo[1,2-c]imidazole. Eur. J. Med. Chem..

[27] Zhang M., Ding Y., Qin H.-X., Xu Z.-G., Lan H.-T., Yang D.-L., Yi C. (2020). One-pot synthesis of substituted pyrrole–imidazole derivatives with anticancer activity. Mol. Divers..

[28] Papadopoulos E.P. (1973). Reactions of pyrrole with isothiocyanates. Preparation and reactions of N-ethoxycarbonyylpyrrole-2-thiocarboxamide and 2-thiopyrrole-1,2-dicarboximide. J. Org. Chem..

[29] Katritzky A.R., Singh S.K., Bobrov S.J. (2004). Novel Synthesis of Bicycles with Fused Pyrrole, Indole, Oxazole, and Imidazole Rings. J. Org. Chem..

[30] Kong W.-J., Chen X., Wang M., Dai H.-X., Yu J.-Q. (2018). Rapid Syntheses of Heteroaryl-Substituted Imidazo[1,5-a]indole and Pyrrolo[1,2-c]imidazole via Aerobic C2–H Functionalizations. Org. Lett..

[31] McNab H., Tyas R.G. (2007). A Thermal Cascade Route to Pyrroloisoindolone and Pyrroloimidazolones. J. Org. Chem..

[32] Trofimov B.A., Stepanova Z.V., Sobenina L.N., Mikhaleva A.I., Ushakov I.A. (2004). Ethynylation of pyrroles with 1-acyl-2-bromoacetylenes on alumina: A formal ‘inverse sonogashira coupling. Tetrahedron Lett..

[33] King H.D., Meng Z., Denhart D., Mattson R., Kimura R., Wu D., Gao Q., Macor J.E. (2005). Enantioselective Synthesis of a Highly Potent Selective Serotonin Reuptake Inhibitor. An Application of Imidazolidinone Catalysis to the Alkylation of Indoles with an alpha,beta-Disubstituted alpha,beta-Unsaturated Aldehyde. Org. Lett..

[34] Schmitz W.D., Denhart D.J., Brenner A.B., Ditta J.L., Mattson R.J., Mattson G.K., Molski T.F., Macor J.E. (2005). Homotryptamines as Potent and Selective Serotonin Reuptake Inhibitors (SSRIs). Bioorg. Med. Chem. Lett..

[35] Toda N., Tago K., Marumoto S., Takami K., Ori M., Yamada N., Koyama K., Naruto S., Abe K., Yamazaki R. (2003). A Conformational Restriction Approach to the Development of Dual Inhibitors of Acetylcholinesterase and Serotonin Transporter as Potential Agents for Alzheimer’s Disease. Bioorg. Med. Chem..

[36] Denhart D., Ditta J., King D., Macor J., Marcin L., Mattson R., Meng Z. (2004). Compounds for the Treatment of Premature Ejaculation.

[37] Karila D., Freret T., Bouet V., Boulouard M., Dallemagne P., Rochais C. (2015). Therapeutic Potential of 5-HT 6 Receptor Agonists. J. Med. Chem..

[38] Tsunoda T., Sasaki O., Itô S. (1990). Aza-Claisen rearrangement of amide enolates. Stereoselective synthesis of 2, 3-disubstituted carboxamides. Tetrahedron Lett..

[39] Hadimani M.B., Mukherjee R., Banerjee R., Shoman M.E., Aly O.M., King S.B. (2015). Ring expansions of acyloxy nitroso compounds. Tetrahedron Lett..

[40] Hashimoto T., Naganawa Y., Maruoka K. (2009). Stereoselective Construction of Seven-Membered Rings with an All-Carbon Quaternary Center by Direct Tiffeneau-Demjanov-type Ring Expansion. J. Am. Chem..

[41] Donslund B.S., Jessen N.I., Bertuzzi G., Giardinetti M., Palazzo T.A., Christensen M.L., Jørgensen K.A. (2018). Catalytic Enantioselective [10+4]-Cycloadditions. Angew. Chem. Int..

[42] Uno H., Imai T., Harada K., Shibata N. (2020). Synthesis of highly functionalized 12-membered trifluoromethyl heterocycles via a nondecarboxylative Pd-catalyzed [6+ 6] annulation. ACS Catal..

[43] Van Putten R.-J., Van der Waal J.C., de Jong E., Rasrendra C.B., Heeres H.J., de Vries J.G. (2013). Hydroxymethylfurfural, A Versatile Platform Chemical Made from Renewable Resources. Chem. Rev..

[44] Lewkowski J. (2001). Synthesis, chemistry and applications of 5-hydroxymethyl-furfural and its derivatives. Arkivoc.

[45] Kuster B.F.M. (1990). 5-Hydroxymethylfurfural (HMF). A Review Focussing on its Manufacture. Starke.

[46] Escobar N., Laibach N. (2021). Sustainability check for bio-based technologies: A review of process-based and life cycle approaches. Renew. Sustain. Energy Rev..

[47] Ahmad F.B., Kalam M.A., Zhang Z., Masjuki H.H. (2022). Sustainable production of furan-based oxygenated fuel additives from pentose-rich biomass residues. Energy Convers. Manag. X.

[48] Leitner W., Lercher J.A. (2014). From Biomass to Fuels: Homogeneous and Heterogeneous Approaches. Chem. Ing. Tech..

